# Should preoperative computed tomography be routine examination for cervicofacial space infections?

**DOI:** 10.1186/s12879-022-07545-6

**Published:** 2022-06-22

**Authors:** Jiayu Liang, Linli Jiang, Maoye Li, Lei Liu, Hui Li

**Affiliations:** State Key Laboratory of Oral Diseases and National Clinical Research Center for Oral Diseases, Department of Oral and Maxillofacial Surgery, West China Hospital of Stomatology, Sichuan University, Chengdu, China

**Keywords:** Cervicofacial, Infection, Odontogenic, Computed tomography, Reoperation

## Abstract

**Background:**

Cervicofacial space infections are potentially life-threatening, which require accurate diagnosis, early incision, and adequate drainage. The utilization of computed tomography (CT) in cervicofacial space infections has significantly increased for its advantages in the evaluation of abscesses, its availability, and low cost. However, the clinical value of preoperative CT imaging in cervicofacial space infections remains controversial for its poor specificity, radiation exposure, potential complications, and extra cost. We, therefore, investigated whether CT examination should be used as a routine examination in the treatment of patients with cervicofacial space infections.

**Methods:**

A retrospective study of all patients affected by cervicofacial space infections that received incision and drainage surgery from Jan 2016 to Dec 2020 was performed at West China Hospital of Stomatology at Sichuan University. Patients were divided into two groups: the group with preoperative CT and without preoperative CT. Outcomes, including reoperation rate, missed diagnosis rate, days of symptom relief, length of stay, duration of surgery, and total cost of hospitalization, were analyzed.

**Results:**

Out of n = 153 patients, 108 patients underwent surgery with preoperative CT and 45 patients without preoperative CT. The reoperation rate in the preoperative CT group (6/108, 5.6%) was significantly lower (P = 0.00) than that in the group without preoperative CT (10/45, 22.2%). Significant reduction of missed diagnosis rate (P = 0.00), days of symptom relief (P = 0.01), length of stay(P = 0.03), and duration of surgery (P = 0.01) were detected in the preoperative CT group. The results demonstrated that the utilization of preoperative CT can reduce the missed diagnosis rate and repeated surgery complications.

**Conclusions:**

We recommend preoperative CT as a routine examination in cervicofacial space infections.

## Introduction

Cervicofacial space infections refer to infections in potential spaces and fascial planes of the head and neck. Streptococcus pyogenes, Staphylococcus aureus, and Klebsiella pneumonia are the predominant bacterial causative agents of cervicofacial space infections [1–3]. Odontogenic infections are common factors in the development of this severe disease [4, 5]. However, infections of deep fascial spaces of the head and neck are often underestimated and misdiagnosed. If not treated properly, the infections will further spread within a short period time, leading to life-threatening complications, such as airway obstruction, mediastinitis, septic embolism, and dural sinus thrombosis [6, 7]. The mortality rates can go up to 38% when complications occur [8, 9].

Cervicofacial space infections require prompt and accurate management [1, 4, 10]. Accurate diagnosis, early incision, and adequate drainage are the main treatment modalities once the abscess has formed. Accurate diagnosis is a prerequisite for effective treatment without complications. However, accurate diagnosis of the cervicofacial space infections is challenging based only on history and clinical examination findings, mainly due to diffuse, ill-defined facial swelling without characteristic clinical manifestations. Previous studies have found that clinical examination alone can only accurately identify abscesses in 33–76% of cases [11, 12]. Underdiagnosis may cause the subsequent spread of the infection, leading to repeated surgery, prolonged hospitalization, and increased total cost. Some surgeons, therefore, resort to the following adjunctive techniques: (1) puncture, which can only be used as a confirmatory diagnosis of cervicofacial infections for its operational blindness, presenting a risk of underdiagnosis; (2) ultrasound (US), which is an option for diagnosis of superficial soft tissue infections of the head and neck, however, inapplicable for deep cervicofacial spaces infections due to the interference of the maxillary bones; (3) magnetic resonance imaging (MRI), which can offer excellent soft-tissue contrast resolution, but is less accessible and requires longer acquisition time and greater patient cooperation [13, 14].

In recent years, the utilization of computed tomography (CT) for diagnosis of cervicofacial space infections has significantly increased for its advantages in aiding in identifying the location and extent of the abscess involved within a short acquisition time [14, 15]. However, some surgeons still question the clinical significance of CT in the treatment of cervicofacial space infections. A study by Rosenthal et al. [16] identified that CT yielded a poor specificity (40%) for the detection of a deep maxillofacial abscess. Vural et al. [17] also reported that the overall accuracy of CT is 63% in identifying abscesses in deep neck infections. Furthermore, patients’ concerns regarding radiation exposure, potential complications, and extra cost have also increased. Currently, there exists a wide variation in the utilization rate of preoperative CT (8–83%) in the treatment of cervicofacial space infections [18–20]. In a study conducted by Sharma et al. [20] in London, only 8% of patients admitted for cervicofacial infections had preoperative CT. In Utah, USA, Nicholas et al. [19] recorded that 69% of patients with neck infections had primary CT. In a study conducted in Florida, USA, the application rate of CT in odontogenic infections reached 83% [18]. Whether preoperative CT should be taken as a routine examination for cervicofacial space infections remains in debate. Until now, there are no large sample cohort clinical studies that have systematically investigated the clinical value of preoperative CT imaging for cervicofacial space infections.

Thus, the purpose of this study was to retrospectively investigate whether preoperative CT should be recommended as routine examination for cervicofacial space infections.

## Methods

To address the research purpose, the authors designed and implemented a retrospective cohort study that was approved by the Research Ethics Board of the Ethics Committee, West China Hospital of Stomatology, Sichuan University with the reference number WCHSIRB-D-2021-056. Patients with cervicofacial space infections, and who presented at our department from Jan 2016 to Dec 2020, were included in this retrospective study. Patients were divided into 2 groups: the group with preoperative CT and the group without preoperative CT. All CT scans were performed on a high-resolution CT scan with three-dimensional reconstructions (Philips MX16 EVO CT, Netherlands), and all scans were obtained at 120 kV, 7700mAs, scanning time of 17.5 s, sections thickness of 1.0 mm thickness.

The inclusion criteria were as follows: (1) patients with a final diagnosis of cervicofacial space infections, defined as infections involving one or multiple spaces including the pterygomandibular, submasseteric, submandibular, submental, sublingual, deep temporal, infratemporal, parapharyngeal, and pretracheal spaces; (2) received surgical incision and drainage.

Patients with the following conditions were excluded: (1) history of head and neck trauma or malignancy near or at the site of the chief complaint; (2) history of chemoradiotherapy; (3) incomplete records (data missing for five or more variables).

### Data collection

The following data were recorded: age, gender, presenting symptoms, etiology, involved space(s) in head and neck, admission WBC (white blood cell count), history of diabetes mellitus, tracheotomy received before surgery, “red flag” signs, reoperation rate, missed diagnosis rate, days of symptom relief, length of stay, duration of surgery, and total cost of hospitalization.

### Study variables

The primary predictor was preoperative CT. The primary outcome variables were reoperation rate and missed diagnosis rate. Reoperation refers to that patients underwent repeated surgery for incision and drainage. A missed diagnosis was determined when one or more spaces in preoperative diagnosis were not diagnosed compared to the final diagnosis.

The other variables related to the study were days of symptom relief, length of stay, duration of surgery, and total cost of hospitalization. The authors take it as symptom relief if there is an improvement when patients’ temperature < 37.2 ℃ or significant reduction of swelling and pain in the chief complaint area.

### Statistical analysis

All statistical analysis was completed using IBM SPSS Statistics software (version 26.0; IBM; Armonk, NY). The Shapiro-Wilk test showed that the data were normally distributed. The results are expressed as percentages, means, and standard deviations. Pearson chi-Square test and continuity correction chi-square test was used to compare categorical data between the groups. The student’s t-test was utilized to compare continuous variables between the groups. P < 0.05 was considered statistically significant.

## Results

In total, 153 patients with cervicofacial space infections were included in the study: 94 (61.4%) men and 59 (38.6%) women with a mean age of 51.7 ± 17.4 years (range, 4 to 89 year). The demographic characteristics of male and female were shown in Table 1. Patients were divided into two groups: 108 patients underwent surgery with preoperative CT examination and 45 patients underwent surgery without preoperative CT examination.

**Table 1:** Demographic characteristics by gender

Study variables	All	Male	Female	T/χ2	*P-*value
Sample size, n (%)	153 (100%)	94 (61.4)	59 (38.6)	NA	NA
Age (year),	51.7 ± 17.4	50.5 ± 17.2	53.6 ± 17.8	1.05	0.29
mean ± SD					
Preoperative CT, n (%)				0.24	0.62
Yes	108 (70.6)	65 (60.2)	43 (39.8)		
No	45 (29.4)	29 (64.4)	16 (35.6)		
Diabetes mellitus, n (%)				2.81	0.09
Yes	19 (12.4)	15 (78.9)	4 (21.1)		
No	134 (87.6)	79 (61.4)	55 (38.6)		
Pearson χ2 test and Student-t test were used for analysis *NA* not applicable, *SD* standard deviation					

Odontogenic (102 patients, 66.7%) was the most common cause in the preoperative CT group and non-preoperative CT group, respectively (Table 2). In the preoperative CT group, 71 cases were of odontogenic origin, among which bone resorption and destruction at the site of the causative tooth were observed in 60 (84.5%) cases using preoperative CT imaging. The most common clinical symptom was swelling (n = 149, 97.4%), followed by limited mouth opening (n = 136, 88.9%) (Table 3).

**Table 2:** Etiology of cervicofacial space infections

Etiology	All, n (%) n = 153	Preoperative CT, n (%) n = 108	Non-preoperative CT, n (%) n = 45
Odontogenic	102 (66.7)	71 (65.7)	31 (68.9)
Post-extraction	12 (7.8)	10 (9.3)	2 (4.4)
Jaw cyst	7 (4.6)	5 (4.6)	2 (4.4)
Lymphadenitis	6(3.9)	2 (1.9)	4 (8.9)
Mass	5 (3.3)	4 (3.7)	1 (2.2)
Peri-tonsillar abscess	4 (2.6)	3 (2.8)	1 (2.2)
Foreign body	2 (1.3)	1 (0.9)	1(2.2)
Trauma	2 (1.3)	2 (1.9)	0 (0)
Unknown	13 (8.5)	10 (9.3)	3 (6.7)

**Table 3:** Presenting symptom(s)

Variables	All, n (%)	Preoperative CT, n (%)	Non-preoperative CT, n (%)
Swelling	149 (97.4)	106 (98.1)	43 (95.6)
Limited mouth opening	136 (88.9)	98 (90.7)	38 (84.4)
Pain	120 (78.4)	86 (79.6)	34 (75.6)
Dysphagia	30 (19.6)	24 (22.2)	6 (13.3)
Elevated floor of mouth	21 (13.7)	17 (15.7)	4 (8.8)
Dyspnea	14 (9.2)	8 (7.4)	6 (13.3)
Temperature ≥ 38.0 ℃	5 (3.3)	4 (3.7)	1 (2.2)

Most commonly affected space was the pterygomandibular space (n = 91, 59.5%), followed by submandibular space (n = 88, 57.5%). The distribution of the infected spaces is illustrated in Table 4. There are 96 patients with one single cervicofacial space involved, 45 patients with two spaces involved, and 12 patients with more than two spaces.

**Table 4:** Distribution of infected spaces of head and neck

Variables	All, n (%)	Preoperative CT, n (%)	Non-preoperative CT, n (%)
Pterygomandibular	91 (59.5)	65 (60.2)	26 (57.8)
Submandibular	88 (57.5)	63 (58.3)	25 (55.6)
Submasseteric	81 (53.0)	56 (51.9)	25 (55.6)
Parapharyngeal	65 (42.5)	50 (46.3)	15 (33.3)
Submental	32 (21.0)	21 (19.4)	11 (24.4)
Infratemporal	28 (18.3)	19 (17.6)	9 (20.0)
Sublingual	15 (9.8)	10 (9.3)	5 (11.1)
Deep temporal	11 (7.2)	5 (4.6)	6 (13.3)
Pretracheal	7 (4.5)	4 (3.7)	3 (6.7)

A comparison of demographics between the two groups is presented in Table 5. Differences showed no significance in terms of age and gender (P *>* 0.05). Further, there was also no statistical difference in the variables related to the condition of diabetes, admission WBC count > 10 × 10^9^/L, tracheotomy received before surgery, and “red flag” signs (either of dyspnea, elevated floor of mouth, and dysphagia) on admission and involved multiple-space infections. Thus, it can be regarded as comparable between these two groups.

**Table 5:** Comparison of subject demographics between the two groups

Study variables	All	Preoperative CT	Non-preoperative CT	T/χ2	*P-*value
Sample size, n (%)	153 (100%)	108 (70.6)	45 (29.4)	NA	NA
Age (year), mean ± SD	51.7 ± 17.4	53.3 ± 17.2	47.9 ± 17.6	1.75	0.08
Gender, n (%)					
Women	59 (38.6)	42 (38.9)	17 (37.8)	0.02	0.9
Men	94 (61.4)	66 (61.1)	28 (62.2)		
Diabetes mellitus, n (%)					
Yes	19 (12.4)	14 (13.0)	5 (11.1)	0.1	0.75
No	134 (87.6)	94 (87.0)	40 (88.9)		
WBC > 10.0 × 10^9^/L, n (%)					
Yes	131 (85.6)	91 (84.3)	40 (88.9)	0.55	0.46
No	22 (14.4)	17 (15.7)	5 (11.1)		
Tracheotomy received before surgery, n (%)					
Yes	11 (7.2)	9 (8.3)	2 (4.4)	0.26^a^	0.61
No	142 (92.8)	99 (91.7)	43 (95.6)		
“Red flag” signs, n (%)					
Yes	61 (39.9)	46 (42.6)	15 (33.3)	1.14	0.29
No	92 (60.1)	62 (57.4)	30 (66.7)		
Involved deep spaces ≥ 2, n (%)					
Yes	57 (37.3)	41 (38.0)	16 (35.6)	0.08	0.78
No	96 (62.7)	67 (62.0)	29 (64.4)		
Pearson χ2 test, continuity χ2 test, and Student-t test were used for analysis “Red flag” signs: either dyspnea, the elevated floor of mouth, or dysphagia presented *NA* not applicable, *SD* standard deviation, *WBC* white blood cell count ^a^Continuity χ2 test					

Of the 153 patients who received surgical incision and drainage, 16 (10.5%) patients underwent secondary surgery. There was a significantly higher proportion of reoperation rate in the group without preoperative CT (10/45, 22.2% vs. 6/108, 5.6%, P < 0.05). When analyzing the missed diagnosis rate, there was a lower rate of missed diagnosis in the group with preoperative CT as compared with that in the group without preoperative CT (11/108, 10.2% vs. 16/45, 35.6%, P < 0.05).

The average days of clinical symptom relief were 2.39 ± 1.34 days in the preoperative CT group and 3.13 ± 1.69 days in non-preoperative group (P *<* 0.05). The mean length of stay for these patients in the preoperative CT group was 10.76 days, whilst the mean length of stay for patients in the non-preoperative CT group was 12.76 days (P < 0.05). Moreover, there was also a statistically significant difference between these two groups in terms of the duration of surgery (48.04 ± 23.53 min vs. 59.33 ± 28.46 min, P *<* 0.05). The average cost of hospitalization for patients in the preoperative CT group is lower than that of the non-preoperative CT group (P > 0.05). A comparison of all study variables between the two groups is presented in Table 6.

**Table 6:** Comparison of treatment outcomes between the two groups

Study variables	All	Preoperative CT	Non-preoperative CT	T/χ2	*P* value
Reoperation, n (%)					
Yes	16 (10.5)	6 (5.6)	10 (22.2)	7.73^a^	0.00^*^
No	137 (89.5)	102 (94.4)	35 (77.8)		
Missed diagnosis, n (%)					
Yes	27 (17.6)	11 (10.2)	16 (35.6)	14.07	0.00^*^
No	126 (82.4)	97 (89.8)	29 (64.4)		
Days of symptom relief, mean ± SD	2.61 ± 1.48	2.39 ± 1.34	3.13 ± 1.69	4.92	0.01^*^
Length of stay(days), mean ± SD	11.35 ± 5.25	10.76 ± 4.94	12.76 ± 5.73	− 2.17	0.03^*^
Duration of surgery(mins), mean ± SD	52.48 ± 28.86	48.04 ± 23.53	59.33 ± 28.46	− 2.54	0.01^*^
Total cost of hospitalization (¥), mean ± SD	13147.65 ± 6829.33	12913.37 ± 6904.07	13709.91 ± 6689.21	− 0.66	0.51
Pearson χ2 test, continuity χ2 test and Student-t test were used for analysis *SD* standard deviation ^a^Continuity χ2 test ^*^Statistically significant					

## Discussion

Cervicofacial space infections are potentially life-threatening and require accurate diagnosis and prompt management. The exact origin, location, and extent of infection are often difficult to define accurately on physical examination alone. Importantly, imaging, including CT, magnetic resonance imaging (MRI), and ultrasound (US), play a major role in the diagnosis and management of cervicofacial space infections [21]. However, these tools have their advantages and disadvantages in diagnostic accuracy, feasibility, and availability [22–24].

Magnetic resonance imaging (MRI) provides superior soft-tissue characterization to CT. In a study by Nurminen et al. [22], the diagnostic accuracy of MRI is 96% in detecting neck infections. According to the study by Babu et al. [23], CT was superior to MRI in the aspects of detection of intralesional gas and calcium and motion artifacts. The drawbacks of MRI are obvious as being time-consuming, expensive, and not readily available, making it impractical for most typical cervicofacial space infections [23, 25].

Ultrasound (US) is an effective imaging modality in identifying the presence of fluid collections and differentiating abscess from cellulitis. Peleg et al. [26] demonstrated the usefulness of ultrasound as a diagnostic tool for superficial fascial space infections. They conducted a study on 50 patients with superficial fascial spaces, in which US helped in identifying abscesses in 22 patients and cellulitis in 28 patients. However, US could not accurately detect those infections involving deep fascial spaces [25, 27], thereby limiting its use in cervicofacial space infections. The study by Kalmovich et al. [24] reported that ultrasound did not correlate well with the presence of parapharengeal abscess. Bassiony et al. [27] found that US can not detect infection of deep facial spaces, including the parapharyngeal, retropharyngeal, masticator, and sublingual spaces.

The advantage of CT is that it clearly shows the surgical anatomical situation, the exact location and extent of extensive, sometimes multi-compartment, inflammatory lesions, and their relationship to the large vessels and the pleural apex [14, 28]. Collins et al. [29] reported that CT(68%) has a higher sensitivity for detecting cervicofacial space infections compared to US (53%), while US has a higher specificity (100%) compared to CT(18%). In this retrospective cohort study, our results demonstrated the significant value of preoperative CT in the treatment of cervicofacial space infections.

Due to complex anatomical location and absence of characteristic clinical manifestations, cervicofacial space infections are often underdiagnosed, which can lead to inadequate drainage and repeated surgery [30, 31]. Reoperation is the most common adverse event after treatment of deep cervicofacial space infections, greatly increasing patients’ physical and mental burden. Sharma et al. [20] and Christensen et al. [32] demonstrated that patients with multiple cervicofacial spaces infections are more likely to undergo reoperation. Thus, our study takes the reoperation rate as the primary variable. A total of 16 out of 153 patients underwent reoperation, making the rate of reoperation in the whole study population 10.5%. There is a significant decrease rate of repeated surgery in the preoperative CT imaged group as compared to the group without preoperative CT (10/45, 22.2% vs. 6/108, 5.6%, P < 0.01). The result showed that preoperative CT examination provides important information in diagnosing cervicofacial space infections, which can guide the treatment course. The results demonstrated patients without preoperative CT are more likely to have repeated surgery for the cervicofacial space infections and uncontrolled inflammation spreading to other adjacent spaces. This highlights the importance of preoperative CT for the treatment of cervicofacial space infections, providing an effective guide for surgical incision and drainage of deep cervicofacial infections.

Odontogenic infections are the most common infections in the head and neck region. The most common odontogenic source of infection was the mandibular molars [4, 33]. Inflammations of the mandibular molars tend to spread to the adjacent spaces, including the pterygomandibular space, submandibular space, and submasseteric space. In the present study, the most frequently occupied spaces were pterygomandibular space and submandibular space, which is consistent with Aliabadi’s study [34].

Accurate diagnosis of deep cervicofacial space infections is the key element for avoiding unnecessary repeated surgery. However, the absence of characteristic clinical presentations often leads to missed diagnosis and delayed treatment. The results showed the rate of missed diagnosis is 35.6% (16/45) in the group without preoperative CT, whereas only 10.2% (11/108) in the group with preoperative CT (shown in Table 6). It is not difficult to find a lower missed diagnosis rate and a correspondingly lower rate of repeated surgery in the preoperative CT imaged group. The results of this study demonstrated that with the aid of CT examinations, the rate of missed diagnosis and the risk of reoperation can be significantly reduced. Therefore, it can be confirmed that preoperative CT plays a crucial role in providing adequate information in managing cervicofacial space infections. Of all the misdiagnosed cases, the most commonly neglected spaces in our study are pterygomandibular and parapharyngeal spaces.

The results also revealed that the use of preoperative CT examinations was significantly effective in improving clinical outcomes of cervicofacial space infections, including the days of symptom relief, the length of hospitalization, and the duration of surgery (shown in Table 6). This further emphasizes the significant value of preoperative CT for defining the precise location and extent of complicated infections and offering an effective medical guide for surgical procedures.

It’s worth emphasizing that CT scans can be used to better assess the extent of osteolytic lesions at the site of odontogenic origin [30]. In the preoperative CT group, 60 cases demonstrated bone resorption and destruction at the site of the causative tooth. This valuable information provided by CT can also prompt the subsequent treatment, such as timely management of the causative tooth.

In the present study, the total cost in patients with cervicofacial space infections was also analyzed. Interestingly, the average total cost of hospitalization did not increase in the group with preoperative CT (shown in Table 6). We speculate that the most likely reason is that the application of preoperative CT shortened the length of stay and minimized the risk of repeated surgery, therefore indirectly decreasing the cost of hospitalization. Similarly, a study by Zawiślak et al. [4] also suggested that accurate diagnosis and treatment of odontogenic infections in head and neck regions can lower treatment costs. We have to consider multiple factors that can influence the total cost of hospitalization. Most of the hospitalization cost is associated with the patient’s condition, while partial hospitalization expenses are independent of the patient’s condition.

Vestibular space infection, palatal space infection, and simple dentoalveolar abscess were not included in this study as it was a retrospective analysis of hospitalized patients with cervicofacial space infections. The majority of these cases present with moderate or severe symptoms when admitted to the hospital. Superficial infections are usually managed in the outpatient department. Besides, US was not available in our hospital from Jan 2016 to Dec 2020 due to the nature of being a specialist unit.

As we know, CRP and PCT are regarded as specific biomarkers for bacterial infection. In recent years, CRP and PCT are of great value in the treatment of cervicofacial space infections because they are used as routine clinical indicators of inflammation [35]. Unfortunately, these indicators between the two groups could not be compared in this study due to the incomplete data.

There were still several limitations in this study, such as the retrospective study design and the results obtained from a single center. Although we advocate that preoperative CT examination should be recommended as a routine examination in the treatment of cervicofacial space infections, a prospective and multiple-center clinical study should be performed.

## Conclusions

This study demonstrated that the missed diagnosis rate and the risk of repeated surgery were significantly reduced in the preoperative CT group. Therefore, we recommend preoperative CT as a routine examination in the treatment of cervicofacial space infections.

## Data Availability

The datasets generated and/or analyzed during the current study are not publicly available due to limitations of ethical approval involving the patient data and anonymity but are available from the corresponding author on reasonable request.

## Abbreviations

CT: Computed tomography
WBC: White blood cell count
